# MART-10 represses cholangiocarcinoma cell growth and high vitamin D receptor expression indicates better prognosis for cholangiocarcinoma

**DOI:** 10.1038/srep43773

**Published:** 2017-03-03

**Authors:** Kun-Chun Chiang, Ta-Sen Yeh, Cheng-Cheng Huang, Yu-Chan Chang, Horng-Heng Juang, Chi-Tung Cheng, Jong-Hwei S. Pang, Jun-Te Hsu, Masashi Takano, Tai C. Chen, Atsushi Kittaka, Michael Hsiao, Chun-Nan Yeh

**Affiliations:** 1 General Surgery Department, Chang Gung Memorial Hospital, Chang Gung University, Keelung, R.O.C, Taiwan; 2 Director of Zebrafish center of Keelung Chang Gung Memorial Hospital, R.O.C, Taiwan; 3 General Surgery Department and Liver research center, Chang Gung Memorial Hospital, Chang Gung University, Kwei-Shan, Taoyuan, R.O.C, Taiwan; 4 Department of Pathology, Chang Gung Memorial Hospital, 222, Mai-Chin Road, Keelung, R.O.C, Taiwan; 5 Genomics Research Center, Academia Sinica, Taipei, R.O.C, Taiwan; 6 Department of Anatomy, College of Medicine, Chang Gung University, Kwei-Shan, Taoyuan, 333, R.O.C, Taiwan; 7 Graduate Institute of Clinical Medical Sciences, College of Medicine, Chang Gung University, Kwei-Shan, Taoyuan, R.O.C, Taiwan; 8 Faculty of Pharmaceutical Sciences, Teikyo University, Sagamihara, Kanagawa, 252-5195, Japan; 9 Boston University School of Medicine, M-1022, 715 Albany Street, Boston, MA 02118, USA

## Abstract

Cholangiocarcinoma (CCA) is a devastating disease due to no effective treatments available. Since the non-mineral functions of vitamin D emerges, 1α,25(OH)_2_D_3_, the active form of vitamin D, has been applied in anti-cancer researches. In this study, we demonstrated that both the 1α,25(OH)_2_D_3_ analog, MART-10, and 1α,25(OH)_2_D_3_ possessed anti-growth effect on human CCA cells with MART-10 much more potent than 1α,25(OH)_2_D_3_. The growth inhibition of both drugs were mediated by induction of G0/G1 cell cycle arrest through upregulation of p27 and downregulation of CDK4, CDK6, and cyclin D3. Human neutrophil gelatinase associated lipocalin (NGAL) was found to be involved in 1α,25(OH)_2_D_3_ and MART-10 meditated growth inhibition for CCA as knockdown of NGAL decreased Ki-67 expression in SNU308 cells and rendered SNU308 cells less responsive to 1α,25(OH)_2_D_3_ and MART-10 treatment. Vitamin D receptor (VDR) knockdown partly abolished MART-10-induced inhibition of NGAL and cell growth in SNU308 cells. The xenograft animal study demonstrated MART-10 could effectively repressed CCA growth *in vivo* without inducing obvious side effects. The IHC examination of human CCA specimen for VDR revealed that higher VDR expression was linked with better prognosis. Collectively, our results suggest that MART-10 could be a promising regimen for CCA treatment.

Cholangiocarcinoma (CCA) accounts for 10–15% of primary liver cancers and is the second most common primary liver cancer after hepatocellular carcinoma. It is estimated that 1/100000 people are diagnosed of CCA per year in the western countries^1^,^2^,^3^,^4^. Of note, the incidence and mortality of CCA has increased in the recent years^5^,^6^. CCA is generally with poor response to traditional chemotherapy and radiotherapy. So far, radical surgery resection remains the best choice of treatment for CCA whenever feasible^7^,^8^,^9^. however, the high recurrent rate after resection and delay diagnosis, which makes most CCA patients not good candidates to receive surgery, lead to poor prognosis^10^. In general, only 25–30% of CCA patients would receive surgery^11^,^12^. Regarding patients with unresectable CCA, prognosis is very dismal with most of them having survival less than 1 year^13^. Thus, to develop a new treatment against CCA should be prioritized.

Since the non-mineral functions of vitamin D has been discovered during the past decades, mainly consisting of pro-differentiation, pro-apoptosis, anti-angiogenesis, etc., vitamin D has emerged as a new regimen against cancer growth and abundances of studies have been published regarding vitamin D application for cancer treatment^14^,^15^,^16^. For clinical application, thousands of 1α,25(OH)_2_D_3_ (the active form of vitamin D) analogs have been synthesized to minimize the side effect of hypercalcemia and to strengthen other effects, mainly the anti-tumor growth effect^17^. To modulate gene expression, 1α,25(OH)_2_D_3_ needs to bind with vitamin D receptor (VDR), which further conjugates with RXR to form a heterodimer^18^. As genes with vitamin D response elements (VDRE) within the promoter area, these genes are subject to 1α,25(OH)_2_D_3_-VDR-RXR complex modulation^19^. So far, at least 693 genes have been found to be 1α,25(OH)_2_D_3_ responsive^20^. Since VDR has been found to exist in a variety of cancer cell lines, it is not surprising that a lot of cancer cells growth are inhibited by 1α,25(OH)_2_D_3_^16^,^21^,^22^,^23^,^24^,^25^. For CCA, overexpression of VDR has been linked to a better prognosis for CCA patients and 22-oxa-1,25-dihydroxyvitamin D_3_, one kind of 1α,25(OH)_2_D_3_ analog, has been shown to be able to repress CCA cell growth *in vitro* and *in vivo*^26^,^27^. In addition, oral supplementation of vitamin D could effectively prevent CCA occurrence and inhibit growth in a chemical-induced rat CCA model^28^. Collectively, application of vitamin D and its analogs to treat CCA seems to be a promising way for CCA treatment under current bleak background.

MART-10 (19-nor-2α-(3-hydroxypropyl)-1α,25(OH)_2_D_3_)^29^, which has 19-nor structure and A-ring modification at C2 position, has been shown by our group to inhibit a variety of cancer cell growth and metastasis^21^,^25^,^30^,^31^,^32^,^33^,^34^ and to be effective and safe in a pancreatic cancer cell xenograft animal model^35^.

Lipocalin-2 (LCN2), known as NGAL as well, is a secreted protein. After secretion, NGAL functions not only as a transporter for some substances and also interacting with other ligands, thus involved in lots of important physiological functions^36^,^37^. NGAL has also been found to play as a oncogene in a myriad of cancers^38^, though some controversies exist^39^,^40^,^41^.

In this study, we aimed to investigate the feasibility of applying MART-10 to treat CCA *in vitro* and *in vivo* and the effect of MART-10 on NGAL expression in CCA. In addition, we would also investigate the relationship between VDR expressions and cliniopathological features of CCA patients to further justify vitamin D and its analogs application in CCA treatment.

## Result

### Anti-proliferative effect of MART-10 and 1α,25(OH)_2_D_3_ on SNU308 and SNU1079 cells

**Figure 1:**
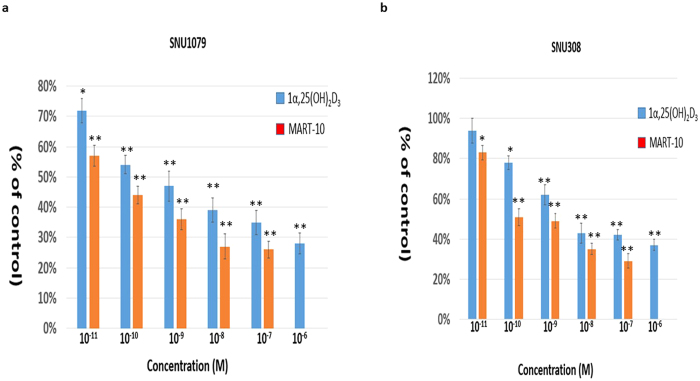
Anti-proliferative effects of 1α,25(OH)_2_D_3_ and MART-10 on CCA cells. Two, four, and six days after plating, cells were treated with 1α,25(OH)_2_D_3_ or MART-10 with indicated concentrations. The cell proliferation was measured by WST-1 method. (**a**) Both 1α,25(OH)_2_D_3_ and MART-10 inhibited SNU1079 cell proliferation dose-dependently with MART-10 much more potent than1α,25(OH)_2_D_3_. (**b**) SNU308 cell proliferation was repressed by 1α,25(OH)_2_D_3_ or MART-10 in a dose-dependent manner. MART-10 was more effective than 1α,25(OH)_2_D_3_. Each value is a mean ± SD of three to five determinations. *p < 0.05, **p < 0.01 versus control.

Figure 1a shows that 1α,25(OH)_2_D_3_, from 10^−6^ to 10^−11^ M, and MART-10, from 10^−7^ to 10^−11^ M, significantly inhibited SNU1079 cell proliferation after 7 days of treatment as determined by WST-1 method. Regarding SNU308 cells, 10^−7^ to 10^−10^ M 1α,25(OH)_2_D_3_ and 10^−7^ to 10^−11^ M MART-10 could effective attenuate cell proliferation (Fig. 1b). Our data clearly indicate that both MART-10 and 1α,25(OH)_2_D_3_ could significantly inhibit CCA cells proliferation with MART-10 much more potent than 1α,25(OH)_2_D_3_.

### Induction of cell cycle arrest at G_0_/G_1_ phase by MART-10 and 1α,25(OH)_2_D_3_ in SNU308 cells

**Figure 2:**
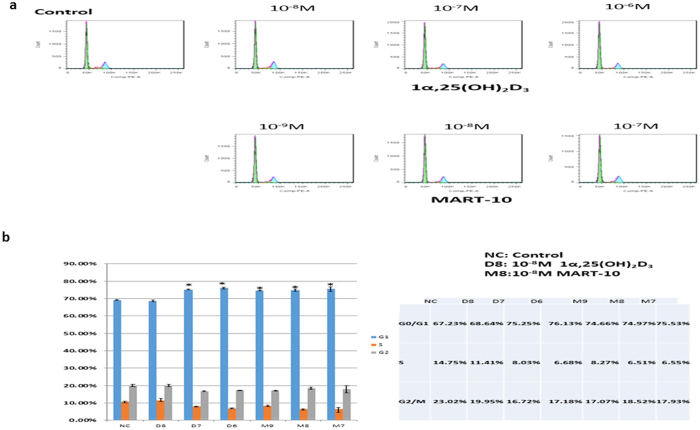
Flow cytometry analysis of cell cycle distribution for SNU308 cells after 1α,25(OH)_2_D_3_ or MART-10 treatment. SNU308 cells were treated with 1α,25(OH)_2_D_3_ from 10^−8^ M to 10^−6^ or MART-10 from 10^−9^ M to 10^−7^ M for two days and then analyzed by flow cytometry. (**a**) A representative DNA histogram for control, 1α25(OH)_2_D_3_- or MART-10-treated SNU308 cells. The total DNA amount of cells (*x*-axis) was obtained by staining cells with propidium iodide. (**b**) The quantitative result of SNU308 cell cycle distribution with or without treatment. Both 1α,25(OH)_2_D_3_ and MART-10 induced G0/G1 arrest in SNU308 cells. (*p < 0.05).

To further understand the growth inhibition mechanisms mediated by MART-10 and 1α,25(OH)_2_D_3_ in CCA cells, we then conducted cell cycle analysis to evaluate the cell cycle distribution by flow cytometry after two days of 1α,25(OH)_2_D_3_ or MART-10 treatment. As shown in Fig. 2, SNU308 cells were treated by 1α,25(OH)_2_D_3_ or MART-10 at concentrations ranging from 10^−8^ to 10^−6^ or 10^−9^ to 10^−7^ M, respectively. Our data suggest that both 1α,25(OH)_2_D_3_ and MART-10 could induced cell cycle arrest at G0/G1 phase in SNU308 cells, leading to the growth inhibition shown in Fig. 1.

### Evaluation of apoptosis induction of 1α,25(OH)_2_D_3_ and MART-10 in SNU308 cells

**Figure 3:**
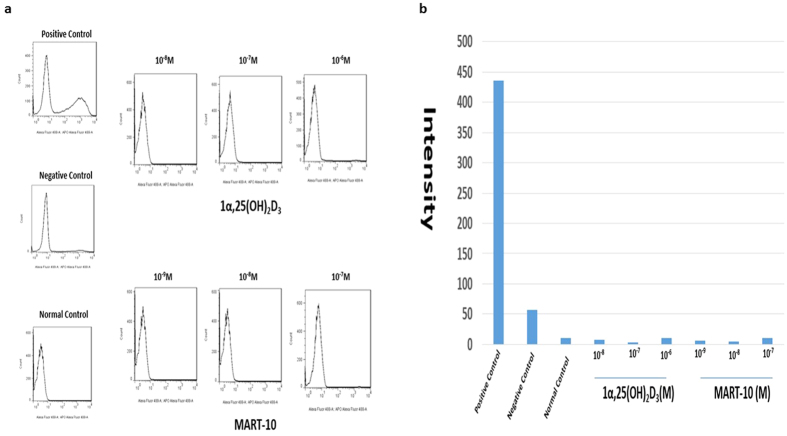
TUNEL assay and Flow cytometry analysis for apoptosis induction in SNU308 cells by 1α,25(OH)_2_D_3_ and MART-10. After two days of treatment, SNU308 cells were stained by APO™-BrdU TUNEL kit and analyzed by flow cytometry to detect apoptosis induction. Our result indicates that neither 1α,25(OH)_2_D_3_ nor MART-10 would induce apoptosis in SNU308 cells.

To evaluate whether 1α,25(OH)_2_D_3_ or MART-10 treatment could induce apoptosis in CCA cells or not, we thus conducted TUNEL assay after two days of 1α,25(OH)_2_D_3_ or MART-10 treatment. As shown in Fig. 3, neither 1α,25(OH)_2_D_3_ nor MART-10 would induce apoptosis in SNU308 cells.

### Evaluation of CDK inhibitors of p21 and p27 expression in SNU308 cells by 1α,25 (OH)_2_D_3_ and MART-10

**Figure 4:**
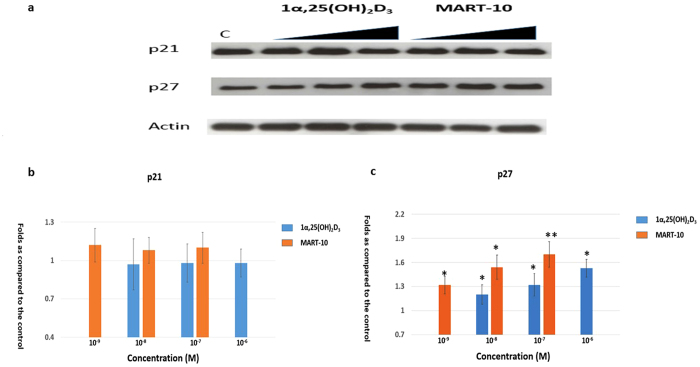
Evaluation of 1α,25(OH)_2_D_3_ and MART-10 effect on p21 and p27 expressions in SNU308 cells. After two days of treatment (10^−6^ to 10^−8^ 1α,25(OH)_2_D_3_ or 10^−7^ to 10^−9^ M MART-10), p21 and p27 expressions were determined by western blot. (**a**) A western blot showing p21 and p27 expression in SNU308 cells with or without treatment. Actin was used as the loading control. (cropped). (**b**) Quantitative result of western blot. Both 1α,25(OH)_2_D_3_ and MART-10 increased p27 expression in SNU308 cells without obvious effect on p21. MART-10 was more effective than 1α,25(OH)_2_D_3_. Each value is a mean ± SD of three to five determinations. *p < 0.05, **p < 0.01 versus control.

To further clarify the mechanisms by which 1α,25(OH)_2_D_3_ and MART-10 arrest CCA cell cycle progression at G_0_/G_1_ phase, the expression of the two main cyclin dependent kinase (CDK) inhibitors, p21 and p27, responsible for G_0_/G_1_ arrest, were examined by western blot analysis (Fig. 4). The quantitative result of wester bot revealed that p27 was upregulated by 10^−6^ to 10^−8^ 1α,25(OH)_2_D_3_ and 10^−7^ to 10^−9^ M MART-10 in a dose dependent manner. As for p21 expression, neither 1α,25(OH)_2_D_3_ nor MART-10 had significant impact in SNU308 cells. Collectively, our result demonstrates that both 1α,25(OH)_2_D_3_ and MART-10 could increase p27 expression SNU308 cells, but not p21, resulting into the G0/G1 cell cycle arrest noted in Fig. 2.

### Evaluation of Cyclin D3, CDK4, CDK6 expression in SNU308 cells after 1α,25 (OH)_2_D_3_ or MART-10 treatment

**Figure 5:**
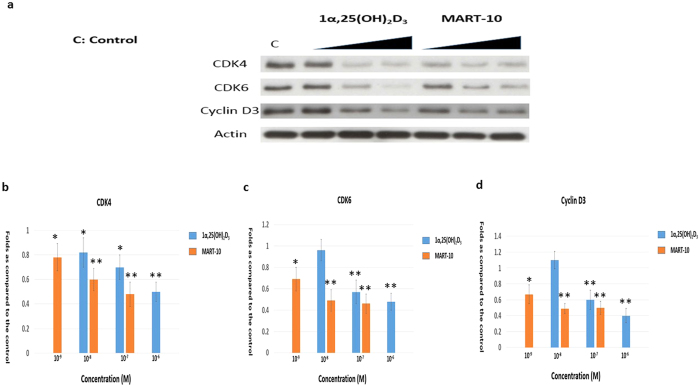
Evaluation of 1α,25(OH)_2_D_3_ and MART-10 effect on CDK4, CDK6, and cyclin D3 expressions in SNU308 cells. After two days of treatment (10^−6^ to 10^−8^ 1α,25(OH)_2_D_3_ or 10^−7^ to 10^−9^ M MART-10), CDK4, CDK6 and cyclin D3 expressions were determined by western blot. (**a**) A western blot showing CDK4, CDK6 and cyclin D3 expression in SNU308 cells with or without treatment. Actin was used as the loading control. (cropped). (**b**) Quantitative result of western blot. Both 1α,25(OH)_2_D_3_ and MART-10 decreased CDK4, CDK6 and cyclin D3 expression in SNU308 cells with MART-10 more effective than 1α,25(OH)_2_D_3_. Each value is a mean ± SD of three to five determinations. *p < 0.05, **p < 0.01 versus control.

We next evaluated CDK4, CDK6, cyclin D3, which are all important elements for cells to pass through G1/S transition checkpoints, expressions in SNU308 cells after two days treatment. As shown in Fig. 5, 1α,25(OH)_2_D_3_ significantly inhibited CDK4, CDK6, and cyclin D3 expressions at 10^−6^ and 10^−7^ M in SNU308 cells; while MART-10, at 10^−7^, 10^−8^, and 10^−9^ M repressed CDK4, CDK6, and cyclin D3 expressions to the greater extent as compared to 1α,25(OH)_2_D_3_ (Fig. 5). Thus, we concluded that SNU308 cell cycle arrest at G0/G1 as induced by 1α,25(OH)_2_D_3_ and MART-10 were also mediated by inhibition of CDK4, CDK6, and cyclin D3 expression in addition to upregulation of p27.

### Evaluation of 1α,25(OH)_2_D_3_ and MART-10 effect on NGAL expression in SNU308 cells

**Figure 6:**
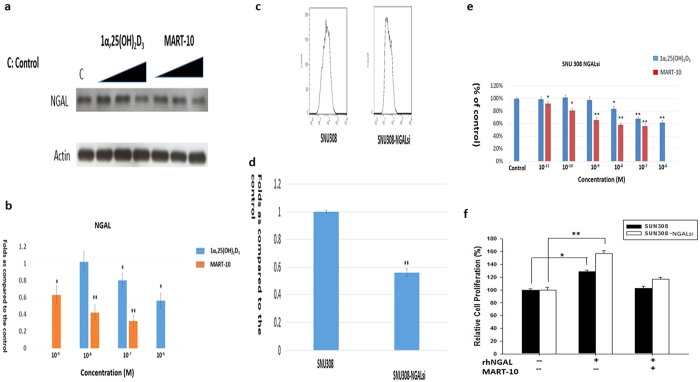
Evaluation of 1α,25(OH)_2_D_3_ and MART-10 effect on NGAL expression in SNU308 cells and proliferation on SNU308 and SNU308- NGALsi cells, and NGAL effect on ki-67 expression of SNU308 cells. NGAL expression in SNU308 cells was evaluated by western blot after two days of 10^−6^ to 10^−8^ 1α,25(OH)_2_D_3_ or 10^−7^ to 10^−9^ M MART-10 treatment. (**a**) A western blot depicting NGAL expression in SNU308 cells with or without treatment. Actin was used as the loading control. (cropped). (**b**) Quantitative result of western blot. Both 1α,25(OH)_2_D_3_ and MART-10 repressed NGAL expression in SNU308 cells with MART-10 more effective than 1α,25(OH)_2_D_3_. To investigate NGAL role in SNU308 cells, Ki-67 expression was measured by flow cytometry in SNU308 cells and SNU308 NGAL-si cells. (**c**) The histogram of Ki-67 expression in SNU308 cells and SNU308 NGAL-si cells. (**d**) The quantitative result of Ki-67 expression in SNU308 cells and SNU308 NGAL-si cells. Our result indicates that knockdown of NGAL in SNU308 cells decreased Ki-67 expression. (**e**) SNU308NGAL-si cells were treated by 1α,25(OH)_2_D_3_ or MART-10 as indicated concentrations every two days. 7 days later, the cell proliferation was measured by WST-1 method. Both 1α,25(OH)_2_D_3_ and MART-10 inhibited SNU308 NGAL-si cells proliferation dose-dependently with MART-10 much more potent than1α,25(OH)_2_D_3._ However, the inhibition effect was less than that observed in SNU308 cells. (**f**) SU308 and SNU308NGAL-si cells were treated by either vehicle, or rhNGAL (0.5 ng/ml), or rhNGAL and MART-10. The cell proliferation was measured two days later. rhNGAL increased cell growth of both SU308 and SNU308NGAL-si cells. The rhNGAL-increased cell growth was abolished by MART-10. (Each value is a mean ± SD of three to five determinations. *p < 0.05, **p < 0.01 versus control).

Previously, we have shown NGAL expression in CCA cells and knockdown of NGAL in CCA cells was able to increase CCA cells doubling time, indicating NGAL role as an oncogene in human CCA^28^. We thus investigated NGAL expression after 1α,25(OH)_2_D_3_ or MART-10 treatment in SNU308 cells. As shown in Fig. 6a and b, a dose dependent manner of inhibition of NGAL in SNU308 cells was observed for 10^−6^ and 10^−7^ M 1α,25(OH)_2_D_3_ and 10^−7^, 10^−8^, and 10^−9^ M MART-10 with MART-10 much more potent than 1α,25(OH)_2_D_3_.

### Evaluation of NGAL effect on Ki-67 expression in SNU308 cells

To further prove the oncogene role of NGAL for human CCA, we next measured Ki-67 expressions in SNU308 cells and SNU308-NGALsi cells by flow cytometry. As shown in Fig. 6c and d, Ki-67 expression in SNU308-NGALsi cells is about 0.56 fold to that of SNU308 cells, implying the oncogene role of NGAL in human CCA and consistent with our previous study^28^.

### Evaluation of 1α,25(OH)_2_D_3_ and MART-10 effect on SNU308 cells and SNU308-NGALsi cells growth

Since NGAL is one of the 1α,25(OH)_2_D_3_ and MART-10 responsive gene in snu308 cells, to further understand NGAL role in 1α,25(OH)_2_D_3_ or MART-10 mediated growth inhibition in human CCA cells, we thus treated SNU308-NGALsi cells with 1α,25(OH)_2_D_3_ or MART-10 and compared the result with that shown in Fig. 1b. As shown in Fig. 6e, 10^−6^, 10^−7^, and 10^−8^ M 1α,25(OH)_2_D_3_ and 10^−7^ to 10^−11^ M MART-10 could effectively repress SNU308-NGALsi cell growth as determined by WST1 method; however, the inhibition effect was much less than that observed in SNU308 cells shown in Fig. 1b. We further applied recombinant human NGAL (rhNGAL) to treat SU308 and SNU308NGAL-si cells. Figure 6f shows that the cell growth of SU308 and SNU308NGAL-si cells were increased by rhNGAL. MART-10 effectively repressed rhNGAL-increased cell growth in both kinds of cells. Our data suggests that the growth inhibition for CCA cells by 1α,25(OH)_2_D_3_ or MART-10 is partly mediated by NGAL inhibition.

### Evaluation of VDR role in MART-10-induced inhibition of NGAL expression and cell growth in CCA cells

**Figure 7:**
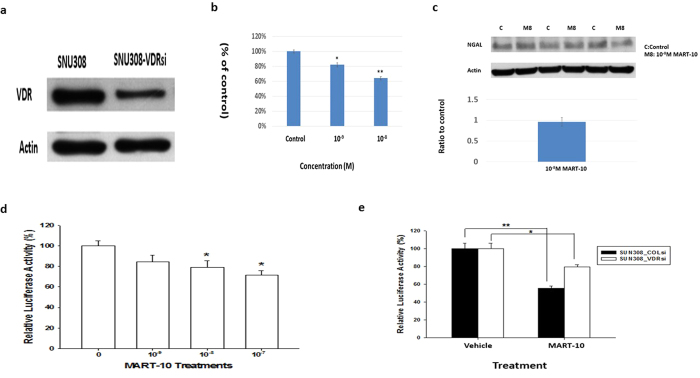
The role of VDR in MART-10-inducd inhibition in cell growth and NGAL expression of CCA cells. (**a**) A western blot depicting VDR expression in SNU308-VDRsi (VDR knockdown) and SNU308-COLsi (mock knockdown) cells. Actin was used as the loading control. (cropped). (**b**) After 7 days of treatment, MART-10 repressed SNU308-VDRsi cell growth, which was less than that noted in SNU308 cells.(*p < 0.05, **p < 0.01). (**c**)The NGAL protein expression in SNU308-VDRsi cells was not influenced by 10^−8^ M MART-10. (**d**) The NGAL reporter activity was inhibited by MART-10 in a dose dependent manner. (*p < 0.05). (**e**) VDR knockdown in SNU308 cells partly abolished MART-10-induced inhibition of NGAL reporter activity. (*p < 0.05 **p < 0.01).

To investigate how MART-10 influenced NGAL expression in CCA cells, we then knocked down VDR in SNU308 cells (SNU308-VDRsi). As shown in Fig. 7a, SNU308-VDRsi cells expressed much weaker VDR expression than SNU308-COLsi cells (mock knockdown of VDR). SNU308-COLsi and SNU308-VDRsi cells were further treated by 10^−8^ and 10^−9^ M MART-10 for 7 days and the proliferation of SNU308-VDRsi cells were inhibited to 64% and 82% as compared to SNU308-COLsi cells (Fig. 7b). NGAL expression in SNU308 cells was not influenced significantly by MART-10 after VDR knockdown (Fig. 7c). The reporter assay indicated that NGAL reporter activity was repressed by MART-10 in a dose dependent manner (Fig. 7d) and NGAL reporter activity was less inhibited by MART-10 as VDR knockdown (Fig. 7e). Based on our result, MART-10 induced NGAL inhibition and MART-10 repressed CCA cell growth partly VDR-dependently.

### Evaluation of 1α,25(OH)_2_D_3_ and MART-10 on CCA cell growth *in vivo*

**Figure 8:**
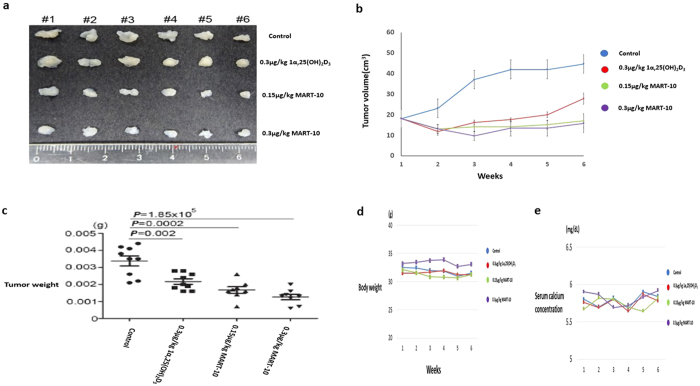
1α,25(OH)2D3 and MART-10 suppress SNU308 cell growth *in vivo* without obvious side effects. After 5 weeks of treatment (two times per week), the xenografted tumors were removed and the tumor weight was measured. Tumor volume, mice body weight and serum calcium were checked every week. (**a**) The photograph to show xenografted tumors from each group. (**b**) Tumor volume change as recorded every week. Both 1α,25(OH)_2_D_3_ and MART-10 repressed xenografted tumor volume significantly as compared to the control with MART-10 groups having more smaller tumor volume than 1α,25(OH)_2_D_3_ group. (**c**) The tumor weights in both 1α,25(OH)_2_D_3_ and MART-10 treated groups are lower than that in the control and MART-10 treated groups had the lowest tumor weight. (**d**) Serum calcium concentration in each group was steady. (**e**) No obvious change of body weight of mice was observed during the experiment period.

To evaluate the effectiveness and safety of application of 1α,25(OH)_2_D_3_ and MART-10 *in vivo*, we next xenografted SNU308 cells in mice and treated them with or without 1α,25(OH)_2_D_3_(0.3 μg/kg) or MART-10(0.15 μg/kg or 0.3 μg/kg) two times a week. The tumor volume, body weight and serum calcium were checked every week. As show in Fig. 8b and c, both 1α,25(OH)_2_D_3_ and MART-10 could significantly repress SNU308 cells growth *in vivo* with MART-10 much more potent than 1α,25(OH)_2_D_3_. The stable body weight and normal serum calcium of mice during the study period indicated both 1α,25(OH)_2_D_3_ and MART-10 are safe under the current applied doses (Fig. 8d and e).

### Correlations between VDR expression and clinicopathological features in CCA patients undergoing hepatectomy

**Figure 9:**
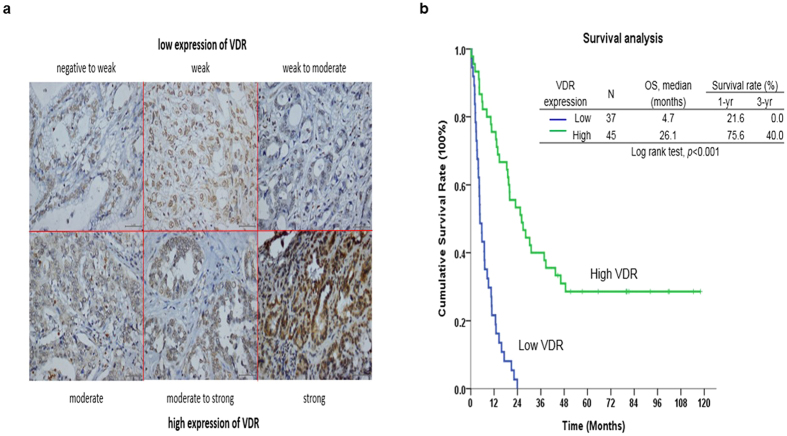
Immunohistochemical stain of VDR in human CCA specimen and the survival analysis of CCA patients based on VDR expression. (**a**) The different intensities of VDR expression in human CCA specimen. (**b**) Kaplan–Meier plot of overall survival in CCA patients undergoing hepatectomy based on their VDR expression levels. The low VDR expression group showed poorer overall survival (*P* < 0.01).

We next analyzed correlations between VDR expression levels and clinicopathological features of 79 CCA patients. Among specimens from 79 CCA patients who underwent hepatectomy, 44 (49.4%) specimens showed high VDR staining intensity (Fig. 9a). Of note, VDR upregulation strongly correlated with negative symptoms, negative lymph node metastasis and a negative resection margin (Supplemental Table 1).

### Low VDR expression is a poor prognostic indicator for CCA patients undergoing hepatectomy

Univariate log-rank analysis was then applied to identify factors that had adverse influences on the overall survival (OS) rate in the aforementioned CCA patients. The presence of positive symptoms, elevated alkaline phosphatase level, elevated CEA levels, a tumor size >5 cm, a positive surgical-margin status and week staining intensity were found to correlate with OS (Supplemental Table 2). However, only low VDR staining intensity was identified as independent predictors for an inferior OS rate in these patients after multivariate Cox proportional hazard analysis (Supplemental Table 3 and Fig. 9b).

## Discussion

Since 1α,25(OH)_2_D_3_ exerts its genomic functions through binding with vitamin D receptor (VDR) and 24-OHase (CYP24A1) is responsible for the degradation of 1α,25(OH)_2_D, the fact that MART-10 has higher VDR binding affinity^42^ and better resistance to CYP24A1-mediated degradation^43^,^44^ leads to the expectable higher VDR transactivation effect of MART-10 than 1α,25(OH)_2_D_3_.

In this study, we showed that MART-10, a C-2 substituted, 19-nor analog of 1α,25(OH)_2_D_3_, possessed much more potent anti-tumor growth effect on human CCA *in vitro* and *in vivo* through induction of cell cycle arrest at G0/G1 phase and inhibition of NGAL. The stable body weight and normal serum calcium concentration noted in the mice during the experiment period (Fig. 8d and e) indicate the safety and non-calcemia characteristic of MART-10 *in vivo*. Since most genomic functions of 1α,25(OH)_2_D_3_ are mediated through binding with VDR, the finding that high VDR expression in human CCA specimen is correlated with better prognosis (Fig. 9 and Supplemental Table 2) further encourages the application of MART-10 in clinical trial for advanced CCA patients.

Cells have to go through cell cycle progression to proliferate. Since cancer cells possess excess mitogenic signalings, which lead to cell cycle dysregulation, cancer cells tend to have unlimited cell proliferation^45^ and targeting cell cycle control becomes one important direction to attenuate cancer cell growth. Our data indicates that both 1α,25(OH)_2_D_3_ and MART-10 could induce G0/G1 cell cycle arrest in SNU308 cells (Fig. 2) with MART-10 more potent than 1α,25(OH)_2_D_3_, leading to the growth inhibition noted in Fig. 1.

The transcriptional factor E2F-1 needs to be active to drive cell through the restriction point to proceed cell cycle progression^46^. However, E2F-1 is inactive in the beginning due to binding with hypophosphorylated retinoblastoma (RB)^47^. To be active, specific cyclins and CDKs are needed to phosphorylate RB, which further releases E2F-1. Among others, CDK4, CDK6, and cyclin D3 are important proteins for RB phosphorylation, which are under negative control by CKIs p21 and p27. As shown in Fig. 4, both 1α,25(OH)_2_D_3_ and MART-10 could upregulate p27 expression in SNU308 cells with MART-10 more effective. No obvious change of p21 expression was observed after treatment in SNU308 cells. In addition, CDK4, CDK6, and cyclin D3 were all downregulated as exposure to 1α,25(OH)_2_D_3_ and MART-10 with the latter more potent (Fig. 5). Our data suggests that the induction of G0/G1 cell cycle arrest in SNU308 cells by 1α,25(OH)_2_D_3_ and MART-10 is mediated by upregulation of p27 and downregulation of CDK4, CDK6, and cyclin D3.

Another important physiological mechanism to maintain tissue homeostasis is apoptosis, which could remove unnecessary cells and curb unlimited proliferation^48^. We investigated whether 1α,25(OH)_2_D_3_ and MART-10 could induce apoptosis in CCA cells. Figure 3 demonstrates that neither 1α,25(OH)_2_D_3_ nor MART-10 could induce CCA cells apoptosis as determined by TUNEL assay.

Human neutrophil gelatinase associated lipocalin (NGAL) has been reported to be expressed in malignant tumors arising from several organs^49^,^50^,^51^. However, the role of NGAL in cancer is tissue-specific. In thyroid cancer, stable silencing of NGAL in thyroid cancer cells leads to a decrease in colony formation *in vitro* and decreased tumorigenecity and tumor size upon subcutaneous injection into nude mice^52^. However NGAL was shown to be involved in the growth and metastasis of endometrial cancer in humans^53^. Regarding CCA, knockdown o NGAL has been shown to increase CCA cells invasiveness and doubling time^28^,^54^. In addition, the serum NGAL levels were found to be significantly elevated in CCA patients compared to those with benign biliary tract disease^55^. Our previous report further indicated that human CCA specimen presented with 66% high NGAL expression^28^. Collectively, NGAL seems to play as an oncogene in human CCA. In this current study, to further verify NGAL role in CCA, the Ki-67 expression was measured in SNU308 cells and SNU308-NGALsi cells. As shown in Fig. 6c and d, SNU308-NGALsi cells has weaker Ki-67 expression than SNU308 cells, consistent with previous studies. As we treated SNU308 cells by 1α,25(OH)_2_D_3_ and MART-10, NGAL expression was decreased with MART-10 more potent (Fig. 6a and b). The less growth inhibition effect of MART-10 and 1α,25(OH)_2_D_3_ on SNU308-NGALsi cells as compared to SNU308 cells and rhNGAL could attenuate MART-10-induced growth inhibition in SNU308 and SNU308NGALsi cells further imply the growth inhibition effect of both drugs on SNU308 is partly mediated by downregulation of NGAL (Fig. 6e and f).

We then next investigated the role of VDR amid MART-10-induced inhibition of NGAL expression and cell growth in CCA cells. Figure 7b indicates that MART-10 repressed SNU308-VDRsi cell growth to a lesser extent as compared to that of SNU308 cells (Fig. 1b). The western blot and reporter assay demonstrated that the reporter activity and protein expression of NGAL in SNU308 cells were less influenced by MART-10 as VDR knockdown (Fig. 7c,d and e). Collectively, we concluded that MART-10 repressed CCA cell NGAL expression in a VDR dependent manner and VDR partly mediated MART-10-induced growth inhibition in CCA cells.

Since most genomic functions of 1α,25(OH)_2_D_3_ are mediated by binding with VDR, we thus investigated VDR expression in human CCA specimens. Previously, Seubwai *et al*. had demonstrated that overexpression of VDR was a good indicator of prognosis for CCA patients^27^. Supplemental Table 1 demonstrates that negative symptoms, negative lymph node metastasis and a negative resection margin were closely correlated with high VDR expression. Regarding OS, after multivariate Cox proportional hazard analysis, only low VDR staining intensity was identified as independent predictors for an inferior OS rate (Supplemental Table 2 and Fig. 8), in line with the previous study^27^.

## Conclusion

CCA is a devastating disease due to resistance to current available chemotherapies and radiotherapy and usually diagnosed late. MART-10, the newly synthesized 1α,25(OH)_2_D_3_ analog, possesses potent anti-growth effect on CCA *in vitro* and *in vivo* without inducing obvious side effect. The finding that high expression of VDR is linked with better OS of CCA patients further implies the feasibility of application of vitamin D in CCA treatment. Collectively, further clinical trial of MART-10 application in CCA treatment is warranted.

## Material and Method

### Vitamin D compounds

1α,25(OH)_2_D_3_ was purchased from Sigma (St. Louis, MO, USA). MART-10 was synthesized as previously described^29^.

### Cell culture

SNU 308 and SNU1079 cells, human CCA cell lines, were obtained from Korean Cell Line Bank (KCLB: 28 Yongon-dong, Chongno-gu, Seoul 110–744, Korea). Cells were grown on RPMI 1640 medium supplemented with 10% FBS and 1% antibiotic-antimycotic agents. Culture medium was changed 3 times per week.

### Cell proliferation assay by WST-1 kit

SNU 308 and SNU1079 cells were plated at about 1,000 cells per cm^2^ in a 48-well cell culture cluster (Costar #3548, Corning Incorporated, Corning, NY, USA). Two days after the initial plating, the cells were treated with ethanol vehicle (control group) or 1α,25(OH)_2_D_3_ or MART-10 at the indicated concentrations. The treatment was repeated 48 hours later. The viable cells were measured by WST-1 kits (Roche #11 644 807 001, Roche Diagnostics, GmbH, Mannheim, Germany) seven days after plating. The assay is based on the cleavage of the tetrazolium salt to formazan by cellular mitochondrial dehydrogenase.

### Flow cytometry for cell cycle analysis

Cells were serum starved for 24 hours and then treated by indicted concentrations of 1α,25(OH)_2_D_3_ or MART-10 for two days. Cells were then processed as previous described^21^,^56^. Cell cycle analysis was performed using FACS-Calibur cytometer and CellQuestPro software (BD Biosciences, San Jose, CA); the data were analyzed using ModFit LT Mac 3.0 software.

### Apoptosis analysis by Flow cytometry

After two days of 1α,25(OH)_2_D_3_ or MART-10 treatment, cell apoptosis was analyzed using a flow cytometer with cells stained by APO™-BrdU TUNEL kit. The operation procedures were according to the manufacture’s guideline.

### Western blot analysis

The detailed procedures for western blot were described previously^21^. The primary antibodies used in this study were monoclonal antibodies against p21 (#2946, Cell Signal, Beverly, MA, USA,), p27 (#3698, Cell Signal), CDK4 (cell signal, #2906, Beverly, MA, USA), CDK6 (cell signal, #3136, Beverly, MA, USA), cyclin D3 (cell signal, #2936, Beverly, MA, USA), NGAL(#PAB9543, Abnova Corporation, Taipei, Taiwan). The secondary antibodies (1:5000) were anti-rabbit (111-035-003, Jackson Immunoresearch, West Grove, PA, USA) or anti-mouse secondary antibodies (Zymed 81–6520). The blots were detected using ECL reagents (WBKLS0500, Millipore, Billerica, MA, USA). Membranes were detected by VersaDoc™ Imaging System (Bio-Rad, Hercules, CA, USA) for analysis.

### Knockdown of NGAL or VDR in SNU 308 cells

The procedures of knockdown of NGAL or VDR in SNU308 cells to obtain SNU308-NGALsi or SNU308-VDRsi cells were described previously^28^.

### Expression of Ki-67 antigen analyzed by flow cytometry

Two days after plating, SNU308 cells and SNU308-NGALsi cells were harvested by trypsin digestion and washed with PBS two times. The detailed procedures were described previously^33^.The final pellets were incubated with Ki67 antibody and analyzed by flow cytometry.

### Reporter Vector Constructs and Reporter Assay

One 7.9 kbp DNA fragment (+2514 to −5450) containing the 5′-flanking region of the human NGAL gene was isolated from the BAC clone (RP11–395P17; Invitrogen, Carlsbad, CA) digested with *Sac I*, and was subcloned into the pGEM3 vector (pGEM3_LCN2). The DNA fragment containing the enhancer/promoter of the NGAL gene (−5450 to +4) was synthesized with primers (T7 and 5′-CCATGGAGTGAGAGGCTCACCTGGGTGG-3′) by PCR using the pGEM3_LCN2 as target DNA, and was digested and cloned into the luciferase reporter vector (pGL3-Basic; Promega Biosciences) at the *Sac I* and *Nco I* sites. Proper ligation and orientation of the reporter vectors were confirmed by extensive restriction mapping and sequencing. The cells were plated onto 24-well plates at 1 × 104 cells/well 1 day prior to transfection. Cells were transiently transfected using the X-tremeGENE HP DNA transfection reagent (Roche Diagnostics, Indianapolis, IN) with 1 μg/well of reporter vector and 0.5 μg/well of the pCMVSPORTβgal (Invitrogen). The luciferase activity was adjusted for transfection efficiency using the normalization control plasmid pCMVSPORTβgal as previously described^57^.

### Animal study

Age-matched, non-obese, diabetic-severe, combined immunodeficient gamma (NOD.Cg-Prkdc^scid^ Il2rg^tm1Wjl^/SzJ JAX^®^, NOD-SCID γ) male mice (8 weeks old, 20–25 g body weight) were used. 5 × 10^6^ of SNU-308 cells were resuspended in 100 μL of PBS and injected into the subcutaneous. All animal experiments were conducted in accordance with a protocol approved by the Academia Sinica Institutional Animal Care and Utilization Committee (IACUC 14-03-665). One week after tumor injection, different treatment were started. Four groups were included in this study, i.e., ethanol treatment group as the shame group (twice per week, intraperitoneal injection, n = 6), 1α,25(OH)_2_D_3_ treatment group (0.3 ug/kg, twice per week, intraperitoneal injection, n = 6), MART-10 low-dose group (0.15 ug/kg, twice per week, intraperitoneal injection, n = 6), and MART-10 high-dose group (0.3 ug/kg, twice per week, intraperitoneal injection, n = 6). The tumor volumes, body weight and blood calcium were measured weekly. Tumor masses were harvested after 5 weeks. Blood calcium was measured using quantitative colorimetric calcium assay kits (BioChain, Newark, CA, USA) according to the manufacturer’s protocol.

### Patient demographics

We examined the demographic features of CCA 79 patients who underwent hepatectomy between 1989 and 2006 at the Department of Surgery of Chang Gung Memorial Hospital, Linkou, Taiwan. The study was approved by the local institutional review board of Chang Gung Memorial Hospital (clinical study numbers 99–2886B, 99–3810B and 102–5813B), and written informed consent for immunohistochemical tumor analysis was obtained from each patient. All methods relating to human experimentation were performed in accordance with the relevant guidelines and regulations.

### VDR immunohistochemistry

VDR expression levels in the aforementioned 79 CCA patients were examined by immunohistochemical staining. Tissue sections (4-μm) prepared from the formalin-fixed, paraffin-embedded hepatectomy specimens were incubated with the primary antibody against VDR (MAB1360 1:1000 dilution; Millipore (Chemicon), USA) at 4 °C overnight. The detailed procedures were described previously^28^. For the assessment of immunohistochemical staining, the percentage of stained target cells was evaluated in 10 random microscopic fields per tissue section (X400 magnification), and their averages were subsequently calculated. Staining intensities were scored as 1 (negative to week), 2 (week), 3 (week to moderate), 4 (moderate), 5 (moderate to strong), or 6 (strong). Specimens with staining intensity scores of ≤3 or >3 were classified as having low or high expression, respectively.

### Statistical analysis

For basic research, the data from each group were compared by unpaired t-test and p value < 0.05 was considered as a significant difference. For animal studies, Mann-Whitney U test was applied. For clinical research, numerical data was compared using independent Student ’ s t -tests. Nominal data was compared using Pearson ’ s χ 2 test or forward stepwise multiple logistic regression, as appropriate. Survival rate was calculated and plotted using the Kaplan-Meier method. Survival analysis was performed by using the log-rank test and multivariate analysis using the Cox proportional hazards model. SPSS statistical software program for Windows (SPSS version 10.0) was employed to conduct the statistics.

## Additional Information

**How to cite this article:** Chiang, K.-C. *et al*. MART-10 represses cholangiocarcinoma cell growth and high vitamin D receptor expression indicates better prognosis for cholangiocarcinoma. *Sci. Rep.* **7**, 43773; doi: 10.1038/srep43773 (2017).

**Publisher's note:** Springer Nature remains neutral with regard to jurisdictional claims in published maps and institutional affiliations.

## Supplementary Material

Supplementary Data
