# Supplementary material for: MART-10 represses cholangiocarcinoma cell growth and high vitamin D receptor expression indicates better prognosis for cholangiocarcinoma
Source: Sci Rep. 2017 Mar 3;7:43773. doi: 10.1038/srep43773 (PMC5335655; doi:10.1038/srep43773)
Supplement: Supplementary Data [file srep43773-s1.pdf]

MART-10 represses cholangiocarcinoma cell growth and high vitamin D receptor expression indicates better prognosis for cholangiocarcinoma

Kun-Chun Chiang[1,2*], Ta-Sen Yeh[3*], Cheng-Cheng Huang[4], Yu-Chan Chang[5], Horng-Heng Juang[#6], Chi-Tung Cheng[3], Jong-Hwei S. Pang[7], Jun-Te Hsu[3], Masashi Takano[8], Tai C Chen[9], Atsushi Kittaka[8], Michael Hsiao[5#] and Chun-Nan Yeh[3#]

[1]General Surgery Department, Chang Gung Memorial Hospital, Keelung, Taiwan, R.O.C, Chang Gung University

[2] Director of Zebrafish center of Keelung Chang Gung Memorial Hospital, Taiwan, R.O.C

[3]General Surgery Department and Liver research center, Chang Gung Memorial Hospital, Kwei-Shan, Taoyuan, Taiwan, R.O.C, Chang Gung University

[4]Department of Pathology, Chang Gung Memorial Hospital, 222, Mai-Chin Road, Keelung, Taiwan, R.O.C

[5] Genomics Research Center, Academia Sinica, Taipei, Taiwan, R.O.C

[6]Department of Anatomy, College of Medicine, Chang Gung University, Kwei-Shan Taoyuan, Taiwan, 333, R.O.C.

[7]Graduate Institute of Clinical Medical Sciences, College of Medicine, Chang Gung University, Kwei-Shan, Taoyuan, Taiwan, R.O.C.

[8] Faculty of Pharmaceutical Sciences, Teikyo University, Sagamihara, Kanagawa, 252-5195, Japan

[9]Boston University School of Medicine, M-1022, 715 Albany Street, Boston, MA 02118, USA

* co-first authors

# co-corresponding authors

Correspondence to: Horng-Heng Juang, Department of Anatomy, Chang Gung University, 259 Wen-Hua 1st Road, Kwei-Shan, Taoyuan 333, Taiwan, R.O.C. TEL: +886-3-2118800; FAX: +886-3-2118112; hhj143@mail.cgu.edu.tw, or Michael Hsiao, mhsiao@gate.sinica.edu.tw or Chun-Nan Yeh, General Surgery Department, Chang Gung Memorial Hospital, 5, Fu-Hsing Street, Kwei-Shan, Taoyuan, Taiwan 333, R.O.C. Tel: 03-3281200-3219; Fax: 03-328581; yehchunnan@gmail.com

Running title: MART-10 represses CCA growth in vitro and in vivo

Keywords: CCA; MART-10; vitamin D; cholangiocarcinoma; 1α,25$(OH)_2D_3$; NGAL; VDR

Table 1 Clinicopathological features between VDR high expression and low expression of cholangiocarcinoma patients

	VDR low expression	VDR high expression	*p*
Age (years)	60.64±9.39	58.92±13.67	0.526
Gender			0.977
Male	15(42.9%)	19(43.2%)	
Female	20(57.1%)	25(56.8%)	
Symptom			**0.006***
Positive	1(2.9%)	11(25.0%)	
Negative	34(97.1%)	33(75.0%)	
AST (IU/l)			0.648
≦34	16(47.1%)	23(52.3%)	
>34	18(52.9%)	21(47.7%)	
ALT (U/L)			0.070
≦36	21(67.7%)	19(46.3%)	
>36	10(32.3%)	22(53.7%)	
ALP (U/L)			0.099
≦94	7(20.6%)	16(38.1%)	
>94	27(79.4%)	26(61.9%)	
Bilirubin (total) (mg/dl)			0.904
≦1.3	29(82.9%)	36(81.8%)	
>1.3	6(17.1%)	8(18.2%)	
Albumin (g/dl)			0.459
≦3.5	12(36.4%)	11(28.2%)	
>3.5	21(63.6%)	28(71.8%)	
Serum CEA (ng/ml)			0.931
≦5	11(44.0%)	14(45.2%)	
>5	14(56.0%)	17(54.8%)	
Size (cm)			0.175
≦5	11(33.3%)	21(48.8%)	
>5	22(66.7%)	22(51.2%)	
Lymph node			**0.015***
Negative	17(48.6%)	33(75.0%)	
Positive	18(51.4%)	11(25.0%)	
Differentiated			0.910
Well	1(2.9%)	1(2.3%)	
Moderate	17(48.6%)	24(54.5%)	
Poorly	16(45.7%)	17(38.6%)	
other	1(2.9%)	2(4.5%)	
Margin			**0.008***
Negative	19(54.3%)	36(81.8%)	
positive	16(45.7%)	8(18.2%)	
Post Chemotherapy			0.526
Without	16(45.7%)	17(38.6%)	
With	19(54.3%)	27(61.4%)	
Post Radiotherapy			1.000
Without	31(88.6%)	38(86.4%)	
With	4(11.4%)	6(13.6%)	

Table 2 Univariate Analysis of Factors Influencing the Overall Survival of the 79 CCA Patients

Factors	Survival Time (months)				*p*
	Median	95% CI of Median	3-year (%)	5-year (%)	
Gender					0.756
Male (n=34)	14.53	6.64-22.42	20.6	17.6	
Female (n=45)	10.82	5.85-15.79	24.4	15.0	
Age					0.547
≦60 (n=39)	12.89	6.05-19.73	25.6	20.5	
>60 (n=40)	12.99	7.23-18.74	20.0	12.0	
Symptoms					**0.006**
Negative (n=12)	37.71	4.78-70.63	58.3	41.7	
Positive (n=67)	10.71	5.83-15.61	16.4	11.8	
AST (IU/l)					0.181
≦34 (n=39)	13.32	9.53-17.10	30.8	22.8	
>34 (n=39)	10.72	3.88-17.56	15.4	10.3	
ALT (IU/l)					0.587
≦36 (n=40)	12.99	6.41-19.56	25.0	19.7	
>36 (n=32)	13.84	5.82-21.86	18.8	9.4	
ALP (IU/L)					**0.008**
≦94 (n=23)	23.90	11.65-36.15	39.1	29.8	
>94 (n=53)	10.29	6.67-13.91	15.1	9.4	
Bil (total) (mg/dl)					0.600
≦1.3 (n=65)	12.99	7.24-18.74	24.6	16.7	
>1.3 (n=14)	10.72	0.00-22.23	14.3	14.3	
Albumin (g/dl)					0.054
≦3.5 (n=23)	5.75	3.95-7.55	17.4	13.0	
>3.5 (n=49)	19.04	13.31-24.76	24.5	15.9	
Serum CEA (ng/dl)					**0.043**
≦5 (n=25)	18.51	2.09-34.93	40.0	27.0	
>5 (n=31)	10.29	4.05-16.53	6.5	6.5	
Margin					**<0.001**
Negative (n=55)	19.43	14.17-24.69	32.7	23.4	
Positive (n=24)	4.41	2.43-6.38	0.0	0.0	
Size					**0.005**
≦5cm (n=32)	19.99	13.75-26.23	37.5	30.7	
>5cm (n=44)	9.11	3.09-15.13	13.6	6.8	
Lymph node					0.099
Negative (n=50)	19.04	8.44-29.63	28.0	17.6	
Positive (n=29)	10.45	0.00-22.71	13.8	13.8	

Histology					0.177
Well (n=2)	2.73	NA	0.0	0.0	
Moderate (n=41)	13.84	7.45-20.23	22.0	17.1	
Poor (n=33)	12.99	5.25-20.72	27.3	17.3	
Others(n=3)	10.72	0.56-20.87	0.0	0.0	
VDR					**<0.001**
Low expression (n=35)	5.66	2.84-8.48	0	0	
High expression (n=44)	26.14	14.99-37.29	40.9	29.2	
Post-op Chemotherapy					0.962
Without (n=33)	5.65	2.47-8.84	30.3	23.9	
With (n=46)	14.53	9.80-19.27	17.4	10.9	
Post-op Radiotherapy					0.074
Without (n=69)	13.32	8.80-17.83	26.1	18.6	
With (n=10)	6.97	5.29-8.65	0.0	0.0	

AST: aspartate aminotransferase; ALT: alanine amionotransferase; ALP: alkaline phosphatase; CEA: carcinoembryonal antigen; CA 19-9: carbohydrate antigen; IU: international unit; op: operation

Table3 Cox's Proportional Hazards Analysis

Factors	Relative Risk (95% confidence interval)	*p*
Symptoms (positive/negative)	2.46(0.55-10.99)	0.238
ALP(>94 g/dl /≦94g/dl)	1.39(0.60-3.26)	0.444
Serum CEA (>5ng/ml/≦5ng/ml)	1.24(0.59-2.58)	0.568
Tumor size (>5 cm/≦5 cm)	1.38(0.66-2.89)	0.397
Margin(positive /negative)	1.54(0.73-3.24)	0.259
VDR expression(high/low)	0.25(0.11-0.53)	<0.001
